# Ozone therapy in colorectal cancer

**DOI:** 10.3389/fonc.2026.1925327

**Published:** 2026-10-30

**Authors:** Yanchu Li, Rong Pu, Sen Yang, Hongxiu Chen, Xiaoxia Zhang

**Affiliations:** 1 Head and Neck Oncology Ward, West China Hospital of Sichuan University, Chengdu, China; 2 Department of Oncology, Chengdu Fuxing Hospital, Sichuan, China; 3 Department of Center for Hepatobiliary Pancreatic Splenic Diseases, Zigong Fourth People’s Hospital, Sichuan, China; 4 West China School of Nursing, Sichuan University; Innovation Center of Nursing Research, Nursing Key Laboratory of Sichuan Province, Chengdu, China

**Keywords:** colorectal cancer, mechanisms, ozone therapy, safety, tumor microenvironment

## Abstract

Colorectal cancer (CRC) remains a leading cause of cancer morbidity and mortality, and metastatic progression, recurrence, chemoresistance, and treatment-related toxicity continue to limit outcomes. Medical ozone has attracted interest as a potential adjunct because, after reacting with biological fluids, it generates short-lived redox mediators such as hydrogen peroxide and lipid oxidation products that may influence NRF2/Keap1, NF-κB, HIF-1α, mitochondrial apoptosis, cytokine signaling, and the tumor microenvironment. However, current CRC-specific evidence is limited and heterogeneous. Experimental studies using CRC cell lines and animal models suggest possible effects on apoptosis, oxidative stress, inflammatory modulation, hypoxia-related pathways, and chemosensitivity, but these findings remain preclinical and are not sufficient to establish antitumor efficacy. Clinical evidence in CRC patients is scarce and mainly relates to supportive care, including radiation-induced rectal injury, chronic treatment-related symptoms, and chemotherapy-induced peripheral neuropathy, rather than tumor response or survival. Importantly, ozone biology is context dependent: activation of adaptive antioxidant responses may protect normal tissues but could also enhance tumor cell stress tolerance under certain conditions. Route-specific safety is also critical, as inhaled ozone is toxic, whereas non-inhalational approaches require standardized dosing and monitoring. Overall, ozone therapy should be considered an investigational adjunct in CRC, with future studies requiring rigorous trial design, biomarker stratification, standardized administration protocols, and clear separation of anticancer endpoints from symptom-control outcomes.

## Introduction

Colorectal cancer (CRC) is one of the most commonly diagnosed malignancies worldwide and remains a major cause of cancer death (1, 2). Contemporary management integrates surgery, chemotherapy, radiotherapy, targeted agents, and immunotherapy in selected molecular subsets, yet metastatic spread, therapeutic resistance, and cumulative toxicity continue to limit durable benefit. Also, CRC biology is tightly linked to oxidative stress, inflammation, and hypoxia-dependent signaling, which together shape proliferation, angiogenesis, immune escape, and treatment response (3). These features have driven interest in interventions capable of modulating the tumor redox environment and microenvironmental stress responses.

Medical ozone(O_3_) has been proposed as one such intervention. O_3_, although recognized as one of the most toxic atmospheric pollutants when inhaled, has emerged as a potential immunomodulatory agent in adjunctive medical therapy in various medical conditions, including cancer (4–7). Administered via parenteral, topical, or injection routes, ozone exerts a hormetic biological response that underlies its therapeutic activity (8, 9). Over the past two decades, its mechanisms of action have been extensively investigated, revealing diverse physiological effects and supporting its proposed use in the management of multiple pathological conditions. In clinical medicine, ozone therapy operates through the induction of controlled oxidative stress generated by ozone–biomolecule interactions. This transient oxidative challenge enhances the endogenous antioxidant defense network and modulates immune function (10). Therapeutic ozone, an allotrope of oxygen, increases tissue oxygenation and exhibits antimicrobial properties, although it has also been shown to induce chromosomal breakages in human cell cultures. The clinical application of ozone therapy was first formalized in 1974 by Wolff, who introduced ozonated autohemotransfusion (O_3_-AHT) as a method of systemic administration (11). Since then, ozone therapy has been increasingly used as an adjunctive intervention in a variety of medical conditions, including oncological disorders (12, 13). However, in oncology, the same adaptive antioxidant programs that might reduce inflammation or protect normal tissue could also, in some contexts, enhance malignant cell survival (5, 14, 15).

This review aims to evaluate the role of ozone therapy in CRC. We summarized proposed mechanisms, preclinical and clinical evidence, and safety.

## Scope and literature basis

This integrative review was designed to evaluate the experimental and clinical evidence relevant to medical ozone therapy in CRC, with particular emphasis on its potential antitumor effects, treatment-modifying effects, supportive-care applications, biological mechanisms, and safety. Because the CRC-specific literature on medical ozone is limited and heterogeneous, a two-level evidence-search strategy was adopted. The primary search focused on studies directly involving colorectal, colon, or rectal cancer models or patients, whereas a supplementary mechanistic search was used to identify studies relevant to redox signaling, inflammation, hypoxia, tissue injury, and other biological pathways necessary for interpretation of the CRC-specific findings. The final eligibility assessment identified 14 full-text reports, five exclusions, and nine studies included in the integrative synthesis.

A structured literature search was conducted in PubMed/MEDLINE and Google Scholar from database inception to May. 2026. The search combined controlled vocabulary, where applicable, with free-text terms relating to medical ozone and colorectal malignancy. For PubMed/MEDLINE, the principal search strategy was (“ozone”[Title/Abstract] OR “ozone therapy”[Title/Abstract] OR “medical ozone”[Title/Abstract] OR “ozonated water”[Title/Abstract] OR “ozone autohemotherapy”[Title/Abstract] OR “major autohemotherapy”[Title/Abstract] OR “rectal ozone”[Title/Abstract] OR “rectal insufflation”[Title/Abstract]) AND (“colorectal cancer”[Title/Abstract] OR “colon cancer”[Title/Abstract] OR “rectal cancer”[Title/Abstract] OR “colorectal neoplasm”[Title/Abstract] OR “colonic neoplasm”[Title/Abstract] OR “rectal neoplasm*”[Title/Abstract])**.

Where appropriate, relevant Medical Subject Headings (MeSH), including terms corresponding to Ozone, Colorectal Neoplasms, Colonic Neoplasms, and Rectal Neoplasms, were incorporated into the search. Google Scholar was searched using combinations of the same concepts, including “ozone therapy colorectal cancer,” “ozone colon cancer,” “ozonated water colon tumor,” “rectal ozone colorectal cancer,” and “ozone chemotherapy colorectal cancer.” A supplementary, targeted mechanistic search was subsequently performed to support interpretation of the CRC-specific evidence. Search terms combined “ozone” or “ozone therapy” with terms including “oxidative stress,” “reactive oxygen species,” “hydrogen peroxide,” “lipid oxidation products,” “NRF2,” “NFE2L2,” “KEAP1,” “NF-κB,” “inflammation,” “IL-6,” “IL-8,” “IL-1β,” “HIF-1α,” “hypoxia,” “apoptosis,” “mitochondria,” “perfusion,” “tumor microenvironment,” “radiotherapy,” “chemotherapy,” “toxicity,” and “adverse events”. In parallel, CRC-specific literature addressing these pathways was consulted when necessary to evaluate whether an ozone-induced biological response could plausibly be beneficial or detrimental in the context of colorectal malignancy.

## Preclinical studies of ozone therapy for colorectal cancer

In 1990, K S Zänker et al. indicated that ozone acts synergistically or at least additive to chemotherapy in the CRC (16). From now on, *in vitro*, ozone has been reported to reduce HT29 cell viability and to augment the effects of 5-fluorouracil and cisplatin, while also suppressing inflammatory cytokines under LPS stimulation (17). In the more recent Colo-320/Colo-741 study, ozone exposure promoted pro-apoptotic signaling, supporting a mitochondrial apoptosis mechanism. Together, these studies provide proof-of-concept that ozone can alter malignant colorectal-cell behavior under experimental condition. Meanwhile, *in vivo*, Kuroda et al. showed that ozonated water increased tumor perfusion and intratumoral oxygenation in colon-26-bearing mice and improved cisplatin efficacy (18). At the same time, gastrointestinal studies indicate that ozone is not biologically inert in normal colonic tissue. Himuro reported that intrarectal ozone caused transient epithelial injury with perturbation of pathways related to DNA replication, cell cycle, and mismatch repair, followed by rapid regeneration (19, 20). It follows that the same intervention may be protective, injurious, or reparative depending on dose, tissue context, timing, and endpoint selection. (**Table 1**).

**Table 1:** Preclinical evidence relevant to colorectal cancer.

Study	Model	Main finding	Interpretation
Simonetti et al (17).	HT29 colon-cancer cells	Ozone reduced viability and modulated inflammatory cytokines; combination with 5-FU/cisplatin showed enhanced effects	Proof-of-concept only
Kendirci-Katirci et al.	Colo-320 and Colo-741 CRC cell lines	Ozone promoted pro-apoptotic signaling and reduced Bcl-2	Supports mitochondrial apoptosis hypothesis
Kuroda et al (18).	Colon-26-bearing mice	Ozonated water improved tumor perfusion/oxygenation and enhanced cisplatin efficacy	Strongest colon-model argument for adjunctive use
Himuro	Mouse colonic epithelium	Transient epithelial injury followed by regeneration after intrarectal ozone	Highlights tissue-context dependence

### Ozone acts as redox signal substance

Ozone does not persist long enough in biological systems to function like a conventional receptor-binding drug. Instead, it reacts rapidly with lipids and other molecules in biological fluids, producing short-lived and longer-lived secondary messengers, especially hydrogen peroxide and lipid oxidation products. These secondary mediators are thought to drive most downstream biological effects. This model is particularly relevant in CRC, where oxidative stress is already deeply involved in tumor initiation, progression, and inflammatory signaling (21). A redox-signaling model accommodates both the observed protective effects in some nonmalignant settings and the pro-apoptotic or chemosensitizing effects reported in some tumor models (22).

### Dual-effective of ozone therapy by KEAP1/NRF2 signaling pathway

Mild oxidative stimulation may activate adaptive defense pathways, whereas higher oxidative stress becomes damaging. Mild ozonization can activate the KEAP1/NRF2-dependent antioxidant response (23). The KEAP1/NRF2 axis is arguably the single most important mechanistic node linking ozone biology to CRC. Ozone-related signaling has been associated with NRF2 activation, which is usually interpreted as beneficial because NRF2 induces antioxidant and cytoprotective programs. Yet in CRC, persistent or excessive NRF2 signaling is not necessarily favorable from an oncologic standpoint. Colon-cancer studies have shown that NRF2 overexpression correlates with tumor-promoting behavior, and experimental inhibition of NRF2-related survival programs can selectively kill NRF2-dependent CRC cells (24). Thus, in normal or injured tissue, ozone-induced NRF2 activation may help restore redox balance and limit treatment-related injury. In tumor cells with constitutively activated antioxidant machinery, the same signaling could theoretically reinforce stress tolerance, promote survival, and blunt therapeutic efficacy. Conversely, if ozone-mediated oxidative perturbation exceeds a tumor cell’s buffering capacity, it may favor apoptosis. Thus, the mechanistic case for ozone in CRC is not a simple ‘NRF2 activation is good’ story, and it is a bidirectional model in which host protection and tumor protection remain competing possibilities.

Tumor cells often live close to the edge of redox tolerance. Additional oxidative stress may either push them toward apoptosis or trigger an adaptive stress response that improves survival. The difference between these outcomes is unlikely to be uniform across CRC molecular subtypes. Therefore, ozone therapy requires careful control of the treatment concentration, just as with the use of arsenic trioxide in the treatment of leukemia.

### NF-κB and inflammatory cytokine signaling

Inflammation is a major driver of CRC biology though NF-κB signal pathway. NF-κB regulates proliferation, survival, angiogenesis, cytokine production, and tumor-promoting interactions between epithelial, stromal, and immune compartments (25, 26). The HT29 study by Simonetti et al. is therefore mechanistically relevant: in that model, ozone exposure, especially under lipopolysaccharide stimulation, was associated with downregulation of key pro-inflammatory mediators such as IL-6, IL-8, and IL-1β (17). That observation should not be exaggerated into a clinical claim, but it is biologically meaningful. IL-6, IL-8, and IL-1β are not incidental inflammatory readouts in CRC; they participate in chemoresistance, tumor-stroma crosstalk, angiogenesis, and immune-cell recruitment. A treatment that dampens these signals could, in principle, shift the microenvironment away from tumor-promoting inflammation.

### Hypoxia, HIF-1α, perfusion, and potential chemosensitization

CRC hypoxia is associated with aggressive behavior, therapy resistance, immune exclusion, and poor prognosis. Recent work has linked elevated HIF-1α signaling to chemoresistance and reduced CD8-positive T-cell infiltration in CRC, reinforcing the importance of hypoxia-responsive pathways in both treatment failure and immune dysfunction (27). The strongest direct support for an ozone-related hypoxia mechanism in a colon-cancer-relevant model comes from Kuroda and colleagues. In colon-26-bearing mice, intraperitoneal ozonated water increased intratumoral blood perfusion and oxygen partial pressure, and it enhanced the antitumor activity of cisplatin. These findings are important because they support an adjunctive rather than stand-alone anticancer role (18).

### Mitochondrial apoptosis and direct tumor-cell injury

Several preclinical observations suggest that ozone can promote mitochondrial apoptotic signaling in colon-cancer models. The HT29 study reported reduced viability with ozone alone and in combination with 5-fluorouracil or cisplatin. More recently, ozone exposure in Colo-320 and Colo-741 CRC cell lines was associated with increased cleaved caspase-3, Bax, and cytochrome c and with decreased Bcl-2 expression, consistent with activation of intrinsic apoptosis pathways (17, 28). Even so, the data do not yet justify any broad claim that ozone is selectively cytotoxic across CRC subtypes. It is molecularly heterogeneous, with marked differences in antioxidant capacity, metabolic wiring, DNA-damage response, and stromal dependence.

### Tumor microenvironment and immune implications

Immune cells, fibroblasts, vasculature, hypoxia gradients, and metabolic competition all influence progression and therapeutic response. Because ozone-related mechanisms intersect with oxidative stress, cytokine balance, perfusion, and cell-death programs, it is reasonable to hypothesize that ozone might also affect the CRC immune microenvironment (29) (**Table 2**). At present, however, this remains mostly speculative in CRC. There is still no convincing evidence that ozone therapy can convert an immune-excluded CRC into an immune-responsive state or improve immunotherapy outcomes. Thus, immune modulation should be presented as a research hypothesis, not as an established effect.

**Table 2:** Proposed mechanistic pathways of ozone therapy in colorectal cancer.

Pathway	Ozone-related effect	Potential oncologic implication	Major caveat
Redox signaling	Generate secondary messengers such as H_2_O_2_ and lipid oxidation products	Reprogramming of oxidative-stress responses	Effects are dose- and context-dependent
Keap1/NRF2	Activation of antioxidant-response signaling	May protect normal tissue and reduce toxicity	May also reinforce tumor survival in NRF2-dependent CRC
NF-κB/cytokines	Reduced IL-6, IL-8, IL-1β in HT29/LPS models	Possible attenuation of tumor-promoting inflammation	Evidence remains cell-line based
Hypoxia/HIF-1α	Improved perfusion and intratumoral oxygenation in animal models	Possible chemosensitization or reversal of hypoxia-driven resistance	No proof yet of reproducible human CRC tumor reoxygenation
Mitochondrial apoptosis	Increased Bax, cytochrome c, cleaved caspase-3; decreased Bcl-2	Possible direct tumor-cell killing	Selectivity across CRC subtypes remains unknown
Tumor microenvironment	Modulation of hypoxia, inflammatory tone, and cell-death patterns	Potential impact on immune contexture	Still largely hypothetical in CRC

## Clinical studies of ozone therapy for colorectal cancer

Based on currently accessible literature, there is no convincing clinical evidence that ozone therapy improves objective oncologic endpoints such as response rate, progression-free survival, or overall survival in CRC. There still lacks the randomized, tumor-specific evidence required to support routine oncologic use (8). However, the available human literature is more credible in supportive-care (**Table 3**).

**Table 3:** Clinical evidence relevant to colorectal cancer.

Study	population	Main finding	Interpretation
Clavo et al. (2013; 2015)	Radiation-induced rectal bleeding/proctitis	Symptomatic improvement reported in refractory cases	Supportive-care evidence, not tumor-control evidence
Clavo et al. (2023)	Symptomatic cancer survivors	Improved HRQOL and reduced toxicity scores	Supports survivorship/toxicity-management interest
OzoParQT/NCT06706544	CIPN trial	Ongoing randomized phase II/III testing rectal ozone for paresthesia	Relevant to CRC survivorship, not direct antitumor efficacy

In previous study, Clavo et al. reported benefit in refractory hemorrhagic radiation proctitis and in persistent radiation-induced rectal bleeding after pelvic radiotherapy. More recently, a study in symptomatic cancer survivors reported significant improvement in health-related quality of life and reduced treatment-related toxicity scores after ozone treatment. However, these are clinically relevant observations, particularly because chronic toxicity is common among CRC survivors, but they should not be misrepresented as proof of direct antitumor efficacy.

A further area of interest is chemotherapy-induced peripheral neuropathy, which is highly relevant to oxaliplatin-based CRC treatment. A 2021 review framed ozone modulation of oxidative stress as the rationale for clinical investigation in Chemotherapy-induced Peripheral Neuropathy (CIPN) (NCT06706544) (30), and an active phase II/III trial is evaluating rectal ozone therapy for paresthesia due to chemotherapy-induced peripheral neuropathy. This line of work is promising for survivorship and symptom management (21).

## Discussion

Although the direct clinical evidence of using ozone in CRC is sparse, the appeal of ozone therapy in CRC lies in its overlap with several core biological problems, including oxidative stress, chronic inflammation, hypoxia, and treatment-related tissue injury (8). Unlike classic cytotoxic agents, ozone is unlikely to act through a single dominant target. Its proposed effects are better understood as network-level modulation of redox-sensitive signaling, perfusion, inflammatory tone, and cellular stress adaptation. This helps explain why the field continues to generate interest even in the absence of definitive clinical validation (3, 22).

A limitation of current research on ozone therapy in CRC is not only simply the scarcity of clinical trials, but also the intrinsically bidirectional nature of ozone-related redox biology (31). Meanwhile, several methodological limitations should be considered when interpreting the current evidence on ozone therapy in CRC. The available literature is characterized by small sample sizes, a paucity of randomized controlled trials, considerable variability in ozone concentration, administration routes, dosing schedules, and treatment duration, as well as substantial heterogeneity across experimental models. These factors limit statistical power, hinder direct comparisons between studies, and reduce the generalizability of preclinical and clinical findings. Moreover, the predominance of *in vitro* studies, animal models, and small observational or supportive-care cohorts precludes robust conclusions regarding clinically meaningful oncologic outcomes, including tumor response, progression-free survival, and overall survival. Differences in tumor models and biological context may further influence sensitivity to ozone-induced redox modulation, which is particularly relevant given the molecular heterogeneity of CRC. In addition, the current evidence base may be affected by publication and selection bias, lack of blinding, selective outcome reporting, and confounding from concomitant anticancer or supportive treatments. Collectively, these limitations underscore the preliminary nature of the existing evidence and highlight the need for adequately powered, prospectively registered randomized studies employing standardized ozone protocols, predefined clinically relevant endpoints, appropriate control groups, and rigorous safety assessment.

Medical ozone rapidly reacts with biological fluids to generate short-lived reactive oxygen species, such as hydrogen peroxide, and longer-lived lipid oxidation products. Within a controlled therapeutic window, these molecules may act as secondary messengers and induce adaptive, hormetic responses. In nonmalignant tissues, this may activate antioxidant and cytoprotective pathways, particularly the KEAP1/NRF2/ARE axis, leading to increased expression of enzymes such as HO-1, NQO1, glutathione-related enzymes, superoxide dismutase and catalase (24, 32). These mechanisms may partly explain reported anti-inflammatory, microcirculatory and tissue-repair effects in non-oncological settings. However, this biology is more problematic in established CRC. NRF2 has a context-dependent role, while it may protect normal or premalignant tissues from oxidative DNA damage, persistent NRF2 activation in cancer cells can enhance survival, detoxification, glutathione synthesis, NADPH regeneration, proteasomal activity and drug-efflux capacity. These effects may increase resistance to chemotherapy and radiotherapy. Therefore, ozone-induced antioxidant signaling could protect normal mucosa but might also benefit tumor cells already dependent on NRF2-mediated redox adaptation. Meanwhile, a similar duality applies to tumor oxygenation. Improved oxygen availability could theoretically enhance radiosensitivity and chemotherapy response, since hypoxia promotes treatment resistance, angiogenesis, epithelial–mesenchymal transition, stemness and immune evasion through pathways involving HIF-1α, VEGF, GLUT1 and CAIX. This is particularly relevant in rectal cancer treated with neoadjuvant chemoradiotherapy. Nevertheless, intermittent or sublethal redox stress may also trigger adaptive tolerance and select for tumor-cell populations with stronger antioxidant defenses. Thus, the effect of ozone is likely to depend on dose, timing, tumor genotype, baseline redox status and tissue context (**Figure 1**).

**Figure 1:**
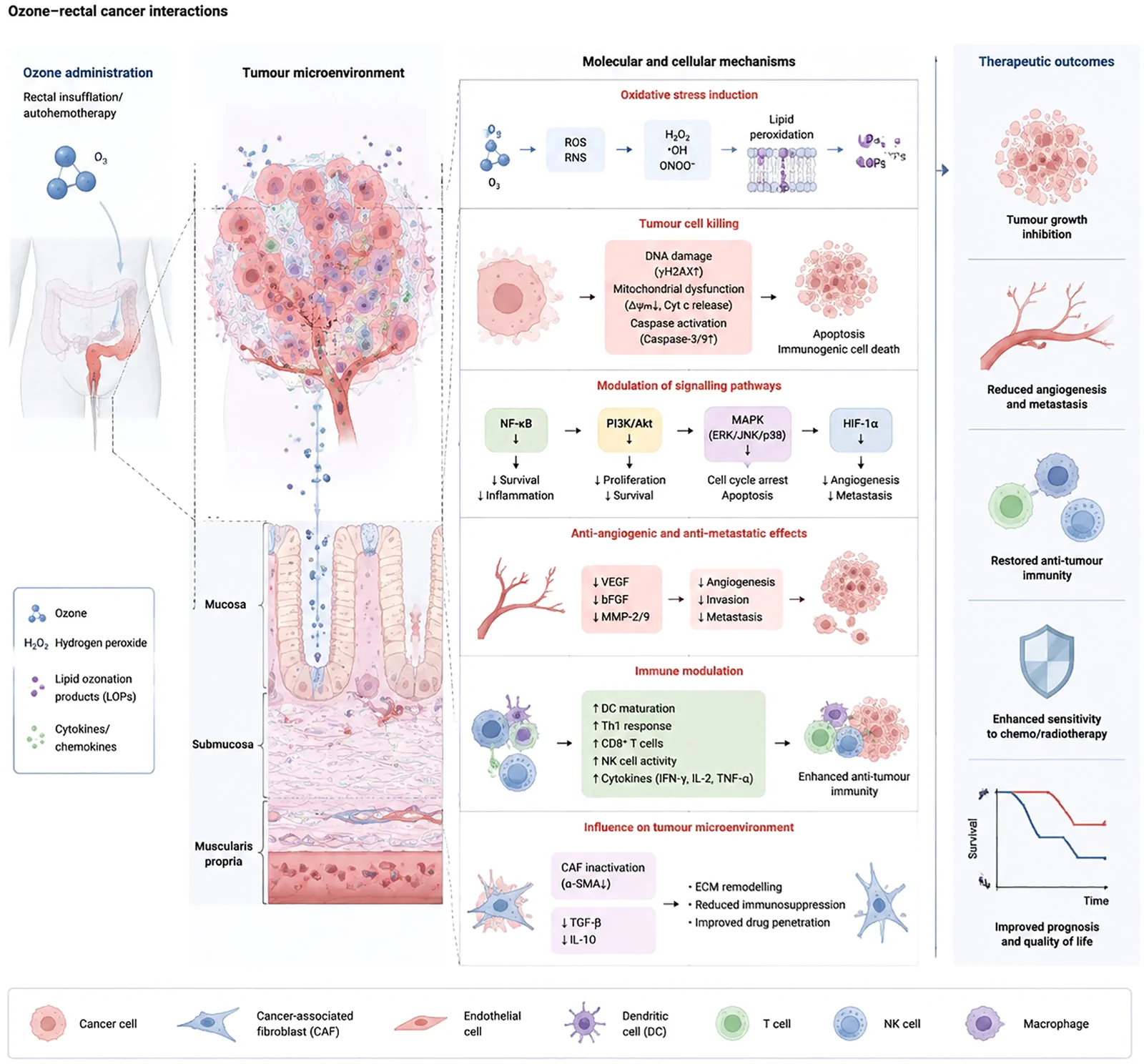
Proposed mechanistic framework of ozone therapy in colorectal cancer. Medical ozone reacts rapidly with biological fluids to generate secondary redox mediators, including hydrogen peroxide and lipid oxidation products. These signals may activate or modulate the KEAP1/NRF2 pathway, NF-κB-associated inflammatory signaling, HIF-1α-dependent hypoxia responses, and mitochondrial apoptosis pathways. In theory, such modulation could reduce inflammatory cytokines, improve tumor perfusion, enhance chemosensitivity, and attenuate treatment-related toxicity. However, the same adaptive antioxidant responses may also protect tumor cells, making the net effect context-dependent and potentially bidirectional.

Future development ought to be biomarker-guided. Relevant stratifiers may include KEAP1/NRF2 pathway status, nuclear NRF2 expression, antioxidant gene signatures such as NQO1, HMOX1, GCLC and GCLM, oxidative-stress markers including GSH/GSSG, 8-OHdG, malondialdehyde and 4-HNE, and hypoxia markers such as HIF-1α, CAIX, GLUT1 and VEGF. Immune-contexture features, including CD8-positive T cells, regulatory T cells, macrophage polarization and Immunoscore, may also be informative. Importantly, different ozone delivery routes should not be treated as interchangeable. Rectal insufflation, ozonated water and major autohemotherapy have distinct exposure sites, pharmacodynamic profiles and safety considerations. Future trials should define route-specific dosing, safety boundaries, biological endpoints and interactions with standard oncological treatments. Overall, ozone therapy may have supportive biological plausibility, but its direct antitumor role in CRC remains unproven and requires mechanism-driven, biomarker-selected and rigorously standardized clinical studies (13, 33).

Safety considerations for medical ozone therapy are fundamentally route-, dose-, and procedure-dependent. Inhalational ozone exposure is unequivocally toxic: as a potent oxidant, ozone reacts with airway lining fluid, epithelial cells, and pulmonary surfactant to generate reactive oxygen species and lipid oxidation products, leading to airway inflammation, epithelial barrier injury, bronchial hyperresponsiveness, and impaired gas exchange (34). U.S. federal regulation likewise identifies ozone as a toxic gas and notes that concentrations required for germicidal activity exceed those safely tolerated by humans or animals; inhalation should therefore not be regarded as an acceptable route of medical administration. This pulmonary toxicity, however, should not be directly extrapolated to controlled non-inhalational approaches, which generate substantially different exposure profiles. Rectal insufflation is most commonly associated with transient bloating, flatulence, and abdominal discomfort, whereas excessive gas volume, rapid insufflation, or high ozone concentrations may increase mechanical or oxidative mucosal injury. Such risks are particularly relevant in CRC, where the bowel may already be ulcerated, inflamed, irradiated, obstructed, or surgically manipulated, potentially increasing susceptibility to bleeding, pain, infection, impaired healing, or perforation. Ozonated water appears to have relatively low local toxicity, although rapid ozone decomposition, variable effective concentrations, mucosal irritation, and aspiration risk complicate exposure standardization. Major autohemotherapy introduces additional risks related to oxidative blood exposure and extracorporeal handling, including venous-access complications, infection, vasovagal reactions, and hemolysis, particularly at excessive oxidative doses or in susceptible individuals such as those with glucose-6-phosphate dehydrogenase deficiency; rare serious complications, including ischemic events and gas embolism, have also been reported. In oncology, ozone-induced redox modulation may additionally interact with chemotherapy, radiotherapy, targeted agents, or immunotherapy. Accordingly, future studies should standardize and report generator characteristics, calibration, gas composition, concentration, flow rate, exposure time, administered dose, route, frequency, and treatment duration, while prospectively collecting adverse events using standardized criteria and predefined stopping rules. Overall, the true incidence of route-specific toxicity remains uncertain because existing studies are generally small, adverse-event reporting is inconsistent, and long-term safety data remain limited.

Indeed, clinical translation of ozone-based interventions remains at an early stage. As of August 2026, the registered ongoing oncology-related studies primarily focus on supportive care and treatment-related toxicities, including chemotherapy-induced peripheral neuropathy, oral mucositis, and radiation-related pelvic toxicity, rather than on direct antitumor efficacy. Two randomized phase II/III trials (NCT04299893 and NCT06706544) are evaluating rectal ozone therapy for persistent chemotherapy-induced peripheral neuropathy, whereas other ongoing or planned studies address oral mucositis, gut microbiome alterations, and refractory treatment-related symptoms; notably, no currently identified active trial is designed to establish an effect on objective tumor response, progression-free survival, or overall survival. Translation of promising preclinical findings is further complicated by fundamental differences between experimental and clinical ozone exposure, since direct ozone–tumor cell contact used *in vitro* does not reproduce the predominantly indirect, secondary-messenger-mediated effects of systemic ozone administration *in vivo*. In addition, the hormetic and concentration-dependent nature of ozone responses, heterogeneity of tumor models and treatment protocols, absence of validated pharmacodynamic markers and a clearly defined therapeutic window, and the complex dual role of redox-responsive pathways such as Nrf2 and NF-κB create uncertainty regarding both efficacy and interactions with ROS-dependent chemotherapy or radiotherapy (8). These scientific uncertainties are reflected in current regulatory and guideline positions. In the United States, FDA regulations remain restrictive regarding medical use of ozone in the absence of demonstrated safety and effectiveness, while regulatory approaches vary internationally; for example, Brazil permits ozone therapy as a complementary procedure, although the oncology indication is not among the uses currently recognized by ANVISA for ozone-emitting medical devices. Major evidence-based oncology supportive-care guidelines, including ASCO guidance for chemotherapy-induced peripheral neuropathy and MASCC/ISOO guidance for cancer therapy-related mucositis, do not currently recommend ozone as standard care. Accordingly, medical ozone should presently be regarded as an investigational adjunct in oncology rather than an established anticancer intervention, pending adequately powered randomized trials that incorporate standardized exposure protocols, oncologic efficacy endpoints, and long-term safety assessment (35).

Above all, the present evidence base has several limitations. Not only colon-cancer-specific studies are few, and many mechanistic inferences come from broader ozone biology or from non-CRC settings, but also available preclinical models vary substantially in route, ozone concentration, exposure time, and biological endpoint. Furthermore, the clinical literature is heterogeneous and largely focused on symptom management rather than tumor outcomes.

## Conclusion

Ozone therapy has a plausible but still unvalidated place in CRC research. But it can be seen that ozone therapy is a potential adjunctive treatment option worthy of further discussion and that may be used in the future for the treatment of CRC. As the field of oncology advances, the integration of ozone therapy with conventional cancer treatments could potentially improve outcomes in patients with CRC. However, at present, there is insufficient evidence to support its routine clinical use, and well-designed randomized controlled trials are still needed.
